# Current understanding of osteoarthritis pathogenesis and relevant new approaches

**DOI:** 10.1038/s41413-022-00226-9

**Published:** 2022-09-20

**Authors:** Liping Tong, Huan Yu, Xingyun Huang, Jie Shen, Guozhi Xiao, Lin Chen, Huaiyu Wang, Lianping Xing, Di Chen

**Affiliations:** 1 Research Center for Computer-aided Drug Discovery, Shenzhen Institute of Advanced Technology, Chinese Academy of Sciences, Shenzhen, 518005 China; 2 Faculty of Pharmaceutical Sciences, Shenzhen Institute of Advanced Technology, Chinese Academy of Sciences, Shenzhen, 518055 China; 3 Department of Orthopedic Surgery, School of Medicine, Washington University in St. Louis, St. Louis, MO 63110 USA; 4 School of Medicine, Southern University of Science and Technology, Shenzhen, 518055 China; 5 Department of Wound Repair and Rehabilitation, State Key Laboratory of Trauma, Burns and Combined Injury, Daping Hospital, Army Medical University, Chongqing, 400042 China; 6 Research Center for Human Tissues and Organs Degeneration, Shenzhen Institute of Advanced Technology, Chinese Academy of Sciences, Shenzhen, 518055 China; 7 Department of Pathology and Laboratory of Medicine, Center for Musculoskeletal Research, University of Rochester Medical Center, Rochester, NY 14642 USA

**Keywords:** Physiology, Pathogenesis

## Abstract

Osteoarthritis (OA) is the most common degenerative joint disease that causes painful swelling and permanent damage to the joints in the body. The molecular mechanisms of OA are currently unknown. OA is a heterogeneous disease that affects the entire joint, and multiple tissues are altered during OA development. To better understand the pathological mechanisms of OA, new approaches, methods, and techniques need to be used to understand OA pathogenesis. In this review, we first focus on the epigenetic regulation of OA, with a particular focus on DNA methylation, histone modification, and microRNA regulation, followed by a summary of several key mediators in OA-associated pain. We then introduce several innovative techniques that have been and will continue to be used in the fields of OA and OA-associated pain, such as CRISPR, scRNA sequencing, and lineage tracing. Next, we discuss the timely updates concerning cell death regulation in OA pathology, including pyroptosis, ferroptosis, and autophagy, as well as their individual roles in OA and potential molecular targets in treating OA. Finally, our review highlights new directions on the role of the synovial lymphatic system in OA. An improved understanding of OA pathogenesis will aid in the development of more specific and effective therapeutic interventions for OA.

## Introduction

Osteoarthritis (OA) is the most common debilitating disease, a leading cause of disability, and is characterized by chronic pain and whole arthropathies such as articular cartilage damage, synovitis, subchondral bone remodeling and osteophyte formation.^1–3^ The prevalence of OA is increasing steadily due to the aging population and worldwide obesity epidemic, which brings a great social burden and imposes a major challenge on public health.^4–6^ It is estimated that 303 million adults in the world were affected, and in China, approximately 61.2 million people had OA in 2017.^7^ Despite the high prevalence, no disease-modifying drugs are currently available. The prescription drugs recommended by international guidelines for OA management merely provide pain relief for symptoms, and the long-term use of these drugs is often associated with significant side effects and toxicities.^8,9^

OA is a heterogeneous and complicated disease that affects multiple joints, such as the knee, hip, lumbar facet joint, and temporomandibular joint (TMJ).^10,11^ The risk factors involved in knee and hip OA include genetics, aging, sex (female), race, occupation (physical labor), obesity, hypertension, abnormal joint strength lines, poor muscle strength, high-intensity exercise, and a history of joint injury.^12–18^ These systemic susceptibility factors and local factors can cause abnormalities in signaling pathways and the related regulatory networks of key functional molecules that transmit pain signals and regulate chondrocyte homeostasis, survival and death and ultimately lead to joint pain and pathological cartilage changes in the synovial joint in OA. The pathological mechanisms of OA are currently unknown. Epigenetic regulation is a newly emerging area associated with alterations in catabolic and anabolic gene expression in osteoarthritic chondrocytes.^19^ Due to the lack of satisfactory management for OA-associated pain, the mechanisms of OA-associated pain and related signaling pathways remain unclear.^20–22^ Recent findings have provided new insights into the roles of new forms of regulated cell death and the synovial lymphatic system in the pathogenesis of OA.^23–26^ To better understand the molecular mechanisms and identify key target(s) for drug discovery and OA treatment, we need to use novel approaches to comprehensively investigate OA mechanisms using newly developed techniques and methodologies. In this review, we will discuss the current understanding of OA pathogenesis and some new approaches, methods, and techniques that have been used in OA research in recent years. First, we focused on the epigenetic regulation of OA, with a particular focus on DNA methylation, histone modification, and microRNA regulation, which have been implicated in OA, and potential epigenome-based therapeutics for OA, followed by a summary of several key mediators in OA-associated pain, including NGF, CGRP, CCL2/CCR2, and TNFα. We then introduced several innovative techniques that have been and will continue to be used in the fields of OA pathology and OA-associated pain, such as CRISPR, scRNA sequencing, and lineage tracing. Next, we discussed cell death regulation in OA pathology, including pyroptosis, ferroptosis, and autophagy, as well as their individual roles in OA and potential molecular targets for treating OA. Finally, our review highlighted new directions on the role of the synovial lymphatic system in OA.

## Epigenetic regulation of osteoarthritis

In addition to genetic regulatory mechanisms, the role of epigenetics has recently drawn increasing attention in the regulation of chondrocyte homeostasis, as well as joint integrity and health. Epigenetic regulation in mammalian cells typically involves DNA methylation, histone modification, and noncoding RNA (miRNA and long noncoding RNA) regulation.^27,28^ Given that epigenetics can govern several signaling pathways simultaneously in a cell-dependent manner, it has been considered a potential therapeutic target to manage OA.

### DNA methylation

**Fig. 1:**
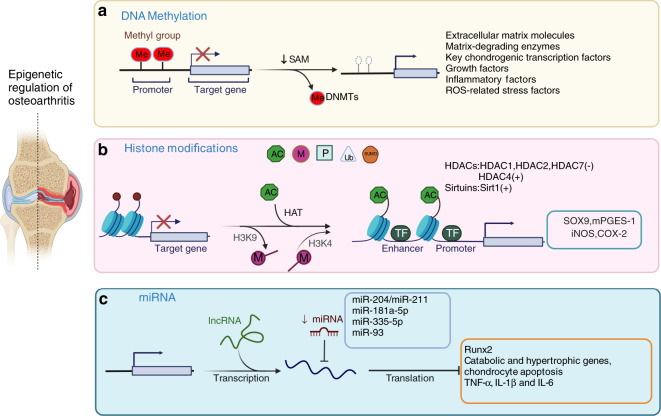
Epigenetic regulation of osteoarthritis. Three types of epigenetic regulation of the molecular pathogenesis of OA. **a** DNA methylation is catalyzed by DNMTs, and abnormal changes in DNA methylation occur in the promoter regions of related genes and signaling pathways in OA chondrocytes. **b** Histone modifications included phosphorylation, methylation, acetylation, ubiquitination, and SUMOylation. Histone acetylation is mainly mediated by HATs, and histone deacetylation is usually catalyzed by two types of enzymes: classic histone deacetylases (HDACs) and sirtuins. Increased H3K4 methylation leads to catabolic responses mediated by iNOS and COX-2 expression in human OA chondrocytes. The methylation of H3K9 increases Sox9 and mPGES-1 expression in human OA chondrocytes. **c** MiR-204/miR-211, miR-181a-5p, miR-335-5p, and miR-93 inhibit Runx2, chondrocyte apoptosis, and the expression of catabolic and hypertrophic genes

DNA methylation usually occurs on the cytosine residues of the dinucleotide sequence CpG. The methylation process includes adding a methyl group to the fifth position of cytosine within a CpG dinucleotide to form 5-methylcytosine (5mC), and this process is mainly catalyzed by DNA methyltransferases (DNMTs) (Fig. 1a). There are three bioactive catalytic DNMTs: DNMT1, DNMT3a, and DNMT3b.^29–31^ DNMT1 serves as a maintenance enzyme that binds hemimethylated DNA to maintain the DNA methylation signatures across the genome during cell division and proliferation. DNMT3a and DNMT3b are considered *de novo* DNMTs since they can create new methylation patterns during development.^29–31^ Several studies have established the crucial role of DNMTs in embryonic development. Global ablation the maintenance enzyme DNMT1 leads to embryonic lethality in mice. Compared to DNMT3a, DNMT3b plays a more imperative role in early embryogenesis, and knockout (KO) of DNMT3b causes embryonic lethality with defects in craniofacial and rib cage development,^32,33^ suggesting a potential regulatory role of DNMT3b in chondrocytes and cartilage development.

In recognition of the roles of DNMTs in chondrocytes, sequencing technology has been used to perform genome-wide DNA methylation analysis has been conducted on healthy individuals and OA patients.^34,35^ Not surprisingly, several well-known pathways and key factors related to chondrocyte homeostasis have alterations in DNA methylation (5mC) in OA patients. For example, a number of studies have shown abnormal changes in DNA methylation in the promoter regions of related genes in OA chondrocytes, including genes encoding extracellular matrix molecules (e.g., Col2A1, CoL9A1, Col10A1, and Acan), matrix-degrading enzymes (e.g., MMP2, MMP3, MMP9, MMP13, Adamts4/5), and key chondrogenic transcription factors (e.g., Sox9, Sox4, and Runx2). Changes in DNA methylation have also been identified in genes related to signaling pathways, including growth factors [e.g., BMP7, sclerostin and growth differentiation factor 5 (GDF5)], inflammatory factors (e.g., IL-1β, IL-8, C-terminal-binding proteins (CtBP), SOCS2 and LEP), and reactive oxygen species (ROS)-related stress factors [e.g., superoxide dismutase (SOD2) and inducible nitric oxide synthase (iNOS)].^36–41^ DNA methylation is a dynamic process involving DNA methylation and demethylation, and the DNA demethylation signature molecule 5-hydroxy-methylcytosine (5hmC) has been identified in OA chondrocytes.^30,42^ The 5hmC signature is generated by the ten–eleven translocation cytosine dioxygenases TET1, TET2, and TET3, which catalyze 5mC oxidation and generate 5mC derivatives, resulting in DNA demethylation at cytosine residues. Among the three bioactive TETs, TET1 is one of the major enzymes responsible for DNA methylation in chondrocytes and the generation of 5hmC in OA chondrocytes. Moreover, in addition to the promoter regions of OA-related genes, irregular CpG methylation in enhancer regions in OA chondrocytes has also been reported.^43–45^ Although further investigation is needed, the data from these studies indicate that DNA methylation can regulate genes in proximity to enhancers in three-dimensional geometry and in such a way that facilitates gene network regulation associated with chondrocyte function, cartilage maintenance, and OA development.

Since alterations in DNA methylation have been found in OA chondrocytes, most recent studies have been focused on examining the underlying regulatory mechanisms and have mainly focused on DNMTs. Zhu et al. recently demonstrated that the expression of DNMT1 and DNMT3a was elevated in OA cartilage from both OA patients and surgically induced murine OA models. Mechanistically, the increases in DNMT1 and DNMT3a resulted in the hypermethylation of the promoter region of peroxisome proliferator-activated receptor-gamma (PPARγ), leading to suppression of PPARγ expression in OA chondrocytes. More importantly, pharmacological inhibition of DNMT1/DNMT3a by 5-azacitidine could demethylate the PPARγ promoter, restore PPARγ expression, and mitigate the severity of cartilage degeneration in mice, suggesting that the DNMT1/3a PPARγ axis is crucial for OA development.^46,47^ In contrast, a reduction in DNMT3b expression was observed in human and murine OA cartilage.^48^ Specifically, the basal expression level of DNMT3b was higher than that of DNMT1 or DNMT3a in healthy chondrocytes and cartilage tissue; however, DNMT3b expression was significantly decreased in murine OA models and OA patients, at least partially due to an increase in the inflammatory (NF-κB) pathway. Comprehensive genome-wide analysis revealed that DNMT3b deficiency led to the induction of 4-aminobutyrate aminotransferase (Abat) through decreased methylation in the gene promoter, which in turn stimulated tricarboxylic acid (TCA) metabolism and mitochondrial respiration in DNMT3b loss-of-function chondrocytes. However, the increases in Abat expression and mitochondrial metabolism could be restored by the overexpression of DNMT3b in chondrocytes, and importantly, DNMT3b gain-of-function induced a chondroprotective effect to maintain homeostasis and integrity of articular cartilage in the knee joint in mice.^48^ Similar to that in the knee joint, the deleterious effect of DNMT3b ablation was also observed in the temporomandibular joint (TMJ). KO of DNMT3b stimulated β-catenin nuclear translocation in TMJ progenitor/stem cells; however, DNMT3b overexpression could normalize Wnt/β-catenin signaling and restore cell homeostasis in vitro.^49^ Overall, recent studies showed the critical role of DNMTs in OA development and suggested that DNMTs may be new molecular targets for OA treatment in the clinic.

### Histone modification

Histone modification is another type of epigenetic regulation in mammalian cells. Histones are alkaline proteins that are typically found in the nucleus and envelop DNA to assemble nucleosomes. Histone modification includes phosphorylation, methylation, acetylation, ubiquitination, and SUMOylation^50,51^ (Fig. 1b). Histone modification usually regulated gene expression by altering chromatin conformation and the ability of transcription factors to access promoter and enhancer regions.^50,51^ Among these histone modifications, methylation and acetylation are the two mechanisms that are extensively studied in OA.^52–61^

The common site for histone methylation is the lysine (K) residues of histone 3 (H3), and recent studies have examined alterations in the epigenetic status of H3 in human OA chondrocytes. On the one hand, it has been reported that the methylation of H3K9 and H3K27 is increased in the promoter region of the key anabolic gene Sox9 and is associated with Sox9 suppression in human OA chondrocytes.^57^ On the other hand, histone methylation has also been shown to be involved in the regulation of the inflammatory response and catabolism in OA development. The Fahmi group evaluated the role of H3K9 and H3K4 methylation in human OA chondrocytes^54,55^ and found that under inflammatory conditions, the recruitment of the histone demethylase LSD1 was enhanced, leading to a reduction in the methylation level of H3K9 in OA chondrocytes, which in turn stimulated the expression of microsomal prostaglandin E synthase-1 (mPGES-1). Moreover, pharmacological blockade of LSD1 could prevent IL-1β-induced H3K9 demethylation and the induction of mPGES-1 in human chondrocytes.^55^ In addition to H3K9 methylation, the Fahmi group investigated H3K4 methylation in human OA chondrocytes. In contrast to H3K9 demethylation, H3K4 methylation was increased by IL-1β in human chondrocytes due to the induced expression of the histone methyltransferase SET‐1A. Increased H3K4 methylation eventually led to catabolic responses mediated by iNOS and COX-2 expression in human OA chondrocytes.^54,62^ These findings suggest that histone methylation is involved in OA pathogenesis through the regulation of anabolic and catabolic activities in chondrocytes.

Similar to histone methylation, histone acetylation and deacetylation have also been extensively investigated in chondrocytes.^52,53,56,58–61^ In mammalian cells, histone acetylation is mainly mediated by histone acetyltransferases (HATs), and histone deacetylation is usually catalyzed by two types of enzymes: classic histone deacetylases (HDACs) and sirtuins.^63^ The expression levels of HDAC1 and HDAC2 are upregulated in OA chondrocytes and are associated with the suppression of anabolic genes, such as Col2a1 and Acan.^52,58,60^ In addition, Huber et al. showed that the expression levels of HDAC1 and HDAC2 were upregulated in OA synovial tissue, indicating an increase in histone deacetylation in joint tissues.^59^ HDAC4 has also been extensively studied, although its function is controversial. Lu et al. demonstrated that the expression of HDAC4 was increased in OA cartilage compared to normal cartilage, and a reduction in HDAC4 levels could significantly suppress the expression levels of *Mmp1*, *Mmp3*, *Mmp13*, and *Adamts4*/*5* in SW1353 chondrosarcoma cells.^61^ However, it has been shown that the expression of HDAC4 is decreased in OA chondrocytes and increases the expression of *Runx2*, *Mmp13*, *Ihh*, and *Col-X*. Therefore, HDAC4 may have chondroprotective effects by inhibiting *Runx2* and other OA-related genes.^53^ Moreover, correlation studies on *Mmp13* and HDAC7 showed that HDAC7 upregulation in OA cartilage was related to enhanced *Mmp13* production and ECM degradation.^56^ Since histone deacetylation is closely correlated with chondrocyte homeostasis and OA, drugs related to HDACs have also been investigated in vitro and in vivo to prevent cartilage degeneration during OA progression. Inhibitors of HDACs, such as trichostatin (TSA) and vorinostat, and HDAC knockdown by small interfering RNAs have been effective in alleviating ECM degradation and cartilage degeneration during OA progression.^64–69^ Furthermore, several siRNAs against HDAC have been approved by the Food and Drug Administration (FDA) in the US for cancer treatment, including vorinostat, romidepsin, valproate and depakote.^66^ Further investigation is needed to determine if HDAC siRNAs could be used for OA treatment.

Significant progress has been achieved in clarifying the role of Sirtuin 1 (SIRT1) in OA pathological progression.^70–74^ Several cellular and animal studies have demonstrated that the SIRT1 protein is found in the nuclei of chondrocytes, as well as in synovial cells. SIRT1 could regulate ECM protein expression in chondrocytes, maintain homeostasis in chondrocytes and cartilage, and play a vital role in inhibiting catabolism, inflammation, oxidative stress, and apoptosis in chondrocytes. SIRT1 expression is decreased in OA chondrocytes.^40,70–74^ Importantly, several studies have demonstrated that the activation of SIRT1 by resveratrol or SRT1720 has chondroprotective effects against cartilage destruction and OA progression. It has been shown that treatment with resveratrol could significantly enhance the gene expression of SIRT1 in chondrocytes, inhibit the IL-1β- or TNFα-induced inflammatory response, and prevent NO-induced apoptosis by regulating Bax and Bcl-2.^75–79^ In addition to resveratrol, SRT1720, another SIRT1 activator, also showed a protective effect against OA development. Intraperitoneal administration of SRT1720 delayed OA progression in mice at least partially by inhibiting the NF-κB pathway, as well as *Mmp13* and *Adamts5* expression, in chondrocytes.^80^ These studies suggest that SIRT1 activation may serve as an ideal therapeutic strategy for OA treatment.

### MicroRNA regulation of osteoarthritis

MicroRNAs (miRNAs) are small, single-stranded RNAs. The average length of a miRNA is 22 nucleotides. MiRNAs negatively modulate target gene expression by binding to the 3′-untranslated region (UTR) of target genes.^81–83^ MiRNAs control approximately 50% of the human transcriptome, and human DNA contains more than 45 000 miRNA target sites.^84,85^ A recent study demonstrated that 142 miRNAs were differentially expressed between damaged and nondamaged OA articular cartilage. To determine the role of miRNA-mediated gene regulation in OA pathology, 238 mRNAs were found to be targeted by differentially expressed miRNAs in OA cartilage using the 2 387 differentially expressed genes as a background. Ten pathways were highly enriched, including the pathways ‘positive regulation of GTPase activity’ and ‘neural development’ associated with nerve growth factor (NGF).^86^ In addition, several miRNAs have been shown to play specific roles in OA progression (Fig. 1c).

Runx2 plays a key role in chondrocyte function.^87^ It has been demonstrated that the deletion of one allele of the *Runx2* gene in *Runx2* global-KO mice or deletion of both alleles of the *Runx2* gene in aggrecan-expressing cells in *Runx2* conditional-KO mice (*Runx2*^*Agc1ER*^) could significantly delay OA progression caused by destabilization of the medial meniscus (DMM) surgery.^88,89^ Our lab has identified that miR-204 and miR-211 are two homologous miRNAs that play key roles in the regulation of Runx2 expression in mesenchymal progenitor cells.^90^ In the joint tissues of aging mice or OA mouse models, the expression of miR-204/miR-211 was significantly decreased compared to that in young mice or sham-operated mice. Deletion of *miR-204*/*miR-211* in *Prx1*-expressing cells (targeting limb mesenchymal progenitor cells) led to progressive OA development in mice.^91^ Significant upregulation of Runx2 protein expression and OA marker genes was observed in *miR-204*/*miR-211* double-KO mice. Deletion of one allele of *Runx2* in a *miR-204*/*miR-211* double-KO genetic background could significantly reverse the OA phenotype observed in *miR-204*/*miR-211* double-KO mice.^91^ These findings suggest that miR-204/miR-211 have chondroprotective effects during OA development. Zhu et al. found that osteoclast-derived factors coupled with exosomal packaging miRNA play a central role in bone formation through paracrine and juxtacrine mechanisms that affect osteoblast differentiation, suggesting that exosome-mediated delivery of miRNA may be a good strategy to treat OA.^92^

MiR-181a-5p is expressed in cartilage in the facet joint (FJ) and is upregulated in human FJ OA and knee OA cartilage. The results were confirmed in the cartilage of rat FJ OA induced by injury and knee traumatic OA. Treatment of rat or mouse chondrocytes with miR-181a-5p antisense oligonucleotides (ASOs) attenuated the expression of marker genes associated with chondrocyte catabolism and apoptosis. MiR-181a-5p ASO treatment reduced cartilage damage, decreased catabolic and hypertrophic gene expression, and inhibited apoptosis and type II collagen degradation in rat FJ OA and mouse knee OA. In addition, the chondroprotective effect of miR-181a-5p ASO was demonstrated in experiments using human OA chondrocytes or ex vivo cartilage explants.^93^

MiRNA-335-5p and miR-93 have anti-inflammatory effects. It has been shown that miRNA-335-5p activates AMP-activated protein kinase (AMPK), which is associated with rapamycin (mTOR) signaling and autophagy activation.^94^ MiRNA-335-5p inhibits OA chondrocyte inflammation by activating autophagy.^95^ MiR-93 attenuates tumor necrosis factor (TNF)-α-, interleukin (IL)-1β- and IL-6-induced inflammatory responses in chondrocytes.^96^ These findings demonstrated that miRNAs are closely associated with the regulation of OA development and progression.

## Osteoarthritis-associated pain

**Fig. 2:**
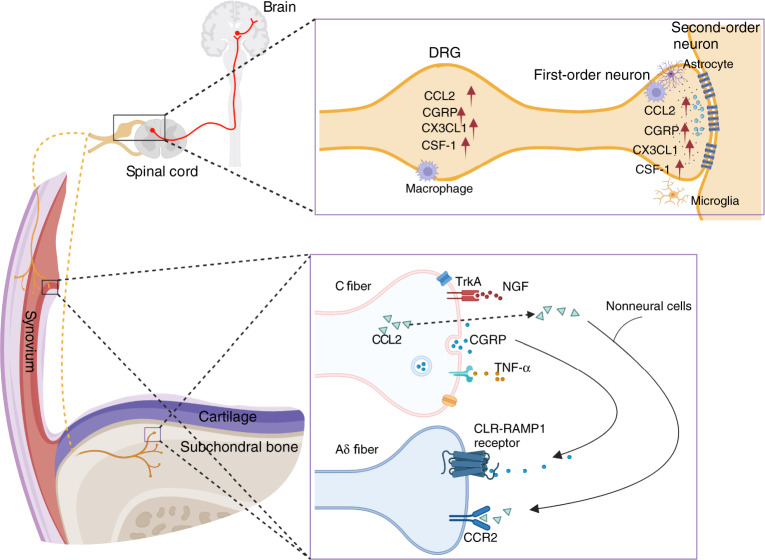
The NGF, CGRP, CCL2/CCR2, and TNFα signaling pathways in OA-associated pain. NGF acts on its receptor TrkA and mediates pain transmission. CGRP is released from C-fiber terminals and binds with calcitonin receptor-like receptor (CLR) and receptor activity-modifying protein 1 (RAMP1) on adjacent Aδ nerve fibers. High expression of chemokines, such as CCL2 and CX3CL1, and other immune mediators, such as colony-stimulating factor 1 (CSF-1) and CGRP, was detected in the DRG and dorsal horn of the spinal cord

Pain is the major symptom that prompts individuals with OA to search for medical assistance. Pain is often associated with joint damage in OA patients. However, pain may not always correlate with structural changes in joint tissues. Sometimes there is a separation between joint damage and pain. OA-associated pain predominantly originates from the synovium and subchondral bone of the joint. Except for normal articular cartilage, which lacks nerve innervation, other tissues in the joint, including the meniscus, ligament, tendon, and fat pad, all have abundant sensory nerve innervation and can be the source of pain. Nociceptors are stimulated by mechanical, chemical or thermal noxious stimuli and transduce these noxious inputs into electrical signals, which are transmitted along the dorsal root ganglion and the dorsal horn of the spinal cord. Ultimately, ascending pathways activate the higher brain center, leading to conscious awareness of pain (Fig. 2). In contrast to fast pain, the persistent inflammatory microenvironment in the OA joint causes peripheral and central nerve sensitization with typical features of mechanical allodynia and hyperalgesia around the joint.^97,98^ Despite the unclear mechanism of nerve sensitization, joint replacement can reverse this paresthesia, suggesting that the central nervous system is highly plastic and that peripheral stimulation may be a major intrinsic factor driving central nerve sensitization.^99^ A better understanding of the mechanisms of OA-associated pain will help in the diagnosis and treatment of OA.

### NGF

Nerve growth factor (NGF) was first identified because it induced the growth of nerves.^100^ We now know that NGF is also associated with intractable pain. NGF and its receptor tropomyosin receptor kinase A (TrkA) are considered new targets for treating OA-associated pain.^101^ Anti-NGF antibodies and TrkA inhibitors have been developed to suppress NGF/TrkA signaling.^102^ Interestingly, local anesthetics can inhibit TrkA.^103^ Fasinumab and tanezumab are humanized monoclonal antibodies against NGF. Patients with symptomatic knee or hip OA were administered gradient doses of fasinumab (1, 3, 6, or 9 mg) in a phase III clinical study.^104^ The results demonstrated that all doses of fasinumab achieved clinically important relief of pain compared with the placebo at 16 weeks. Destructive arthropathy was reported in one patient who received 6 mg fasinumab. The latest phase III clinical study with a 24-week follow-up period showed that tanezumab (5 and 2.5 mg) significantly improved WOMAC pain and function and Patient’s Global Assessment of OA with a 2.8% incidence rate of rapidly progressive OA in the tanezumab 5 mg and 1.4% in tanezumab 2.5 mg groups.^105^ Therefore, the underlying mechanisms of NGF-induced rapidly progressive OA should be carefully and thoroughly examined. It has been reported that increases in the angiogenic factor VEGF, the axonal promoting factor NGF/TrkA and sensory neuronal distribution in individuals with FJ OA and low back pain (LBP) have been detected. These findings were associated with high expression levels of inflammatory cytokines, pain-related molecules, and cartilage-degrading enzymes in the degenerative cartilage of FJ.^106^ Seidel et al. reported that NGF was predominantly distributed in damaged cartilaginous tissues (80%) and in bone marrow (20%) in FJ OA, which was distinct from that in the subchondral bone of knee OA.^107^ NGF and downstream substance P are associated with cartilage pathology in FJ OA. Despite the high risk of joint damage in knee OA, NGF inhibitors have demonstrated good efficacy in treating LBP. A phase III clinical study of NGF antagonists in the treatment of chronic LBP showed that fasinumab improved both LBP and function, and the adverse effect of rapidly progressive OA only occurred in subjects with accompanying peripheral OA.^108^

### CGRP

Calcitonin gene-related peptide (CGRP) is expressed in primary afferent nociceptive unmyelinated C-fibers and is a critical factor in migraine pain.^109^ CGRP is expressed in nociceptive C-fibers. CGRP is released from C-fiber terminals and sensitizes adjacent Aδ nerve fibers.^110^ Aδ nerve fibers express calcitonin receptor-like receptor (CLR) and receptor activity-modifying protein 1 (RAMP1). These findings are consistent with recent observations that the anti-CGRP antibody fremanezumab blocks induced firing of Aδ but not C-fibers.^111^ CGRP receptors were also found in vascular smooth muscle cells and joint tissues innervated by CGRP-positive nerve fibers.^110^ Although the source of OA-associated pain has not been fully defined, the detection of substance P (SP) and CGRP fibers in the synovial tissues in mice suggests that synovial tissue may be the major source of pain during OA development.^112^ In animal studies, intra-articular injection of CGRP caused mechanical allodynia in naïve mice, and CGRP receptor antagonist and CGRP neutralizing antibody treatment alleviated OA-associated pain.^113^ In OA patients, serum CGRP levels and the density of CGRP-positive nerve fibers were highly correlated with OA-associated pain symptoms and disease severity.^114^ However, a phase II clinical trial showed that galcanezumab, a CGRP monoclonal antibody, failed to relieve OA-associated pain.^115^ Interestingly, accumulating evidence has shown sexual dimorphism in chronic pain.^116,117^ Recently, Uchida et al. demonstrated that the expression of CGRP and its receptor RAMP1 in the OA synovium was markedly increased in women compared to men, and CGRP expression was positively correlated with pain severity in females but not in males.^118^ These findings highlight the different pain mechanisms in males and females with OA. Future studies should determine the role of CGRP signaling in OA-associated pain in females.

### CCL2/CCR2

The development of pain behavior is a complicated process involving multiple signaling steps, including changes in the expression and production of key mediators of pain in peripheral organs and the expression of pain mediators in the sensory neurons of the dorsal root ganglia (DRG).^119,120^ Some of this evidence was observed in nerve injury animal models.^121^ It has been shown that chemokines and chemokine receptors, especially chemokine (C-C motif) ligand 2 (CCL2) and its receptor chemokine (C-C motif) receptor 2 (CCR2) mediate pain behavior in the DRG and spinal cord.^119,122^ The role of CCL2/CCR2 signaling in pain mediation during OA development was determined using the DMM OA mouse model. Malfait’s group performed longitudinal studies to monitor pain behaviors and associated molecular mechanisms in the sensory neurons that innervate the knee and demonstrated the role of CCL2/CCR2 in the mediation of pain behavior during OA development.^123^

It is well documented that chemokine–chemokine receptor axes can excite DRG nociceptors by activating molecules such as TRP, and sodium channels have been shown to modulate monocyte/macrophage recruitment in multiple inflammatory diseases.^119,124^ However, the underlying cellular and molecular mechanisms of CCL2/CCR2 signaling in OA-associated pain remain poorly understood. Raghu et al. found that CCL2- and CCR2-deficient mice exhibited reduced OA pathology and low local monocyte/macrophage infiltration in joints.^125^ Blocking CCL2/CCR2 signaling with bindarit, a CCL2 synthesis inhibitor, and RS-504393, a CCR2 antagonist, markedly reduced macrophage accumulation and improved synovitis and cartilage lesions in mouse OA. A recent study demonstrated that CCL2/CCR2 signaling caused knee hyperalgesia through direct activation of CCR2 on sensory afferents in peripheral joints.^126^ Therefore, CCL2/CCR2 signaling involves both peripheral and central mechanisms that contribute to OA-associated pain. Targeting CCL2/CCR2 signaling in the local joint and in the innervating dorsal root ganglia, as well as the dorsal horn, are potential strategies for treating OA-associated pain.

### TNF-α

Inflammation can be a major cause of joint damage and OA-associated pain. Although central nociceptive pathways are involved in OA-associated pain, the interaction of the immune system with nociceptive neurons is critical for inflammatory pain. Following joint damage and peripheral nerve injuries during OA development, neurons release chemokines, such as CCL2 and CX3CL1, and other immune mediators, such as colony-stimulating factor 1 (CSF-1).^127–129^ These factors activate microglia and astrocytes, which can release proinflammatory cytokines, including TNF-α, IL-1β, and IL-6, the chemokine CCL2, excitatory amino acids (EAAs), and nitric oxide (NO).^128,130–132^ Neurons in the dorsal horn of the spinal cord express receptors for many of these inflammatory factors and cytokines, including TNF-α, IL-1β, IL-6, and IL-17.^133,134^ Activating neuronal cytokine receptors could modify neuronal function. For example, it has been shown that TNF-α and IL-1β promote excitatory synaptic transmission and inhibit inhibitory synaptic transmission in neurons in the spinal cord.^135,136^

Previous studies have demonstrated that TNF-α is a major proinflammatory cytokine and is closely associated with OA pathogenesis; however, clinical trials of patients with hand OA did not achieve good results.^137^ In a multicenter study, patients with inflammatory hand OA that flared after NSAID washout were enrolled for anti-TNF-α therapy. Etanercept did not show notable pain relief compared with placebo after 24 weeks of treatment in erosive OA patients.^138^ In the HUMOR trial, subcutaneous injection of adalimumab did not improve VAS pain scores or OA-related pathology, such as synovitis and bone marrow lesions, in patients with erosive hand OA.^139^ TNF-α has two high-affinity and specific receptors: TNFR1 and TNFR2. These two receptors are differentially expressed and seem to have distinct functions. TNFR1 is expressed by nearly all cell types and primarily mediates inflammation, while TNFR2 is expressed by certain cells, such as neural cells and vascular endothelial cells, and inhibits inflammation in inflammatory arthritis and neurodegenerative diseases, as well as cardiac diseases. A recent study demonstrated that TNFR2 is also expressed in chondrocytes and that the activation of progranulin (PGRN)/TNFR2/14-3-3ε signaling in chondrocytes alleviates OA-associated pain and protects against OA.^140^ These findings suggest that TNFR2 signaling is a promising target for treating OA-associated pain and pathology (Fig. 2).

## CRISPR technology

The ability to manipulate genetic information is critical for studying gene function and revealing biological mechanisms. Advances in genome and transcriptome engineering technologies have sparked a new revolution for life science research in the past decade.^141^ CRISPR/Cas systems are derived from the prokaryote adaptive immune system that protects bacteria and archaea against invading genetic materials.^142^ These systems consist of clustered regularly interspaced short palindromic repeats (CRISPR) and multiple genes encoding CRISPR-associated (Cas) proteins that adjoin the CRISPR array.^143^ A diverse range of CRISPR/Cas systems has been used to target and modulate nucleic acids. Whole CRISPR/Cas systems rely on CRISPR RNA (crRNA) or on a single guide RNA (sgRNA) for target recognition. The spacer part of the crRNA or sgRNA hybridizes with the targeting sequence that is upstream of the protospacer adjacent motif (PAM) or protospacer flanking sequence (PFS), and then the Cas cleaves the target DNA or RNA.^144,145^ Thus, by designing crRNA or sgRNA containing proper spacer sequences, CRISPR/Cas systems can achieve cleavage at any locus of interest next to a PAM or a PFS.

**Fig. 3:**
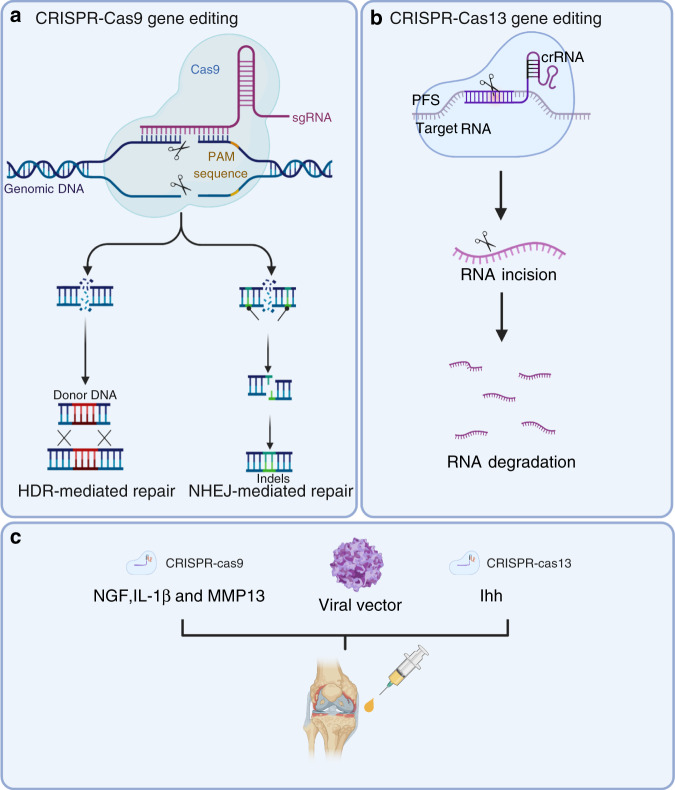
CRISPR/Cas systems have been used for gene editing and treating OA. **a** Cas9 generates DNA double-strand breaks that activate the DNA damage response and induce repair through various endogenous pathways: nonhomologous end joining (NHEJ) or homologous directed repair (HDR). **b** Cas13 cuts the target RNA via intrinsic RNase activity. **c** CRISPR–Cas9-mediated NGF, IL-1β, and MMP13 deletion and CRISPR–Cas13-mediated *ihh* knockout were used for OA treatment

Of the current emergence genome editing tools, the most widely used is the class of RNA-guided endonucleases known as Cas9 (Fig. 3a). Specifically, *Streptococcus pyogenes* Cas9 (SpCas9) was the first to be reprogrammed for genome editing in eukaryotic cells, and it remains the most commonly used Cas9.^146^ Cas9 generates DNA double-strand breaks (DSBs) that activate the DNA damage response and induce repair by the endogenous pathways: nonhomologous end joining (NHEJ) or homologous directed repair (HDR).^147^ NHEJ is an error-prone repair process that leads to the generation of small insertions or deletions (Indels).^148^ Thus, it is useful for silencing gene expression by disrupting the protein reading frame.^149^ HDR is an error-free repair process that corrects pathogenic mutations by using a repair DNA template to stimulate homologous recombination. HDR can be used to introduce the desired change by providing donor DNA that contains a homology sequence to the fractured target site, such as gene site-directed mutation or the insertion of a larger fragment of DNA.^150^ It is important to note that NHEJ is markedly more active than HDR. Therefore, increasing the efficiency of HDR following Cas9-mediated DSBs is widely pursued to fully examine the potential of genome editing to introduce accurate genomic modification.^151,152^

CRISPR/Cas9 technology has been widely used in the construction of plant, animal, and cellular models.^153–155^ A representative example of this is the generation of osteocalcin-knockout rats.^156^ The osteocalcin-null rat models showed increased trabecular bone and improved biomechanics. IL-1β is a proinflammatory cytokine that induces the expression of many genes related to OA, such as tumor necrosis factor-alpha (TNF-α).^157^ It is interesting to note that exposure of human articular chondrocytes (hAC) to TNF-α leads to increased levels of IL-1β expression.^158^ Karlsen and colleagues silenced the IL-1β cytokine receptor (IL1-R1) and simultaneously inserted a puromycin-resistance gene into hACs with CRISPR/Cas9 technology. In their study, unedited cell lines showed increased inflammation when exposed to recombinant IL-1β. Conversely, the IL1-R1-deficient cell line did not cause an obvious inflammatory response.^159^ These models provide a platform for understanding fundamental molecular mechanisms that are related to healthy joint tissue and further exploring potential targets of OA treatment. A particularly inspiring direction is the development of Cas9 as a therapeutic molecule for curing hereditary diseases.^160,161^ Delivery tools for gene-editing molecules include adeno-associated viral vectors, lentiviral vectors, lipid nanoparticles, and other nonviral vectors.^162^ Clinical trials aimed at evaluating the ability of CRISPR/Cas9 to safely correct genetic diseases such as sickle cell disease (NCT03745287), β-thalassemia (NCT03655678), and Leber congenital amaurosis type 10 (NCT03872479) have been initiated. We recently investigated the roles of NGF, IL-1β, and MMP13 in OA development by deleting these genes in joint tissues using the CRISPR–Cas9 technique^163–165^ (Fig. 3c). The key findings of this study were that joint tissue NGF deficiency could efficiently eliminate joint pain but not structural OA damage, and deletion of these three potential harmful OA genes for OA (NGF, IL-1β, and MMP13) had a strong chondroprotective effect, inhibiting pathological damage of joint tissues and alleviating joint pain caused by destabilization of medial meniscus (DMM) surgery.^166^ The flexibility, programmability, and high efficiency of CRISPR/Cas9 technology have promoted genome editing in studies ranging from fundamental science to translational medicine. Perhaps CRISPR/Cas9 could be used to treat OA.

Traditional genomic editing relies on DSB repair pathways, which can cause various side effects in cells, such as large deletions and complex rearrangements around the target, the emergence and expansion of p53-inactivating mutations, and chromosomal truncations.^167–169^ The risks associated with permanent DNA modifications raise serious safety concerns regarding the clinical applications of the CRISPR/Cas9 system. The emergence of the CRISPR/Cas13 family, which cuts a target RNA via intrinsic RNase activity, provides another promising method for gene therapy^145,170^ (Fig. 3b). Currently, type A, type B, type D, type X, and type Y Cas13 family members have been identified.^171,172^ Cas13 targeting has some advantages compared to Cas9; it avoids inducing DNA damage to cells and is theoretically reversible, since it affects RNA. In 2020, Yang and colleagues used CasRx, which is derived from type D, to knockdown *Pten*, *Pcsk9*, and *lncLstr* in mouse hepatocytes and successfully modulate complex metabolic networks.^173^ The researchers also targeted vascular endothelial growth factor A (VEGFA), an angiogenic growth factor, and suppressed choroidal neovascularization (CNV) in a mouse model of age-related macular degeneration (AMD).^174^

It has been reported that overactivation of the Indian hedgehog (Ihh) pathway in chondrocytes strengthens the severity of OA.^175^ However, knockout of the *Ihh* gene is undesirable for OA treatment since it is lethal in animals.^176^ In this context, Cas13-mediated *Ihh* knockdown will be a more desired choice through cartilage-specific delivery in adults. OA-related factors, including matrix metalloproteinases (MMPs), inflammatory cytokines, and growth factors, could be potential targets of the CRISPR/Cas13 system^163,164^ (Fig. 3c).

## Single-cell RNA sequencing

Synovial tissues mainly contain two types of cells: synovial fibroblasts (SFs) and macrophages. During OA initiation, synovial cells interact with macrophages/leukocytes and release inflammatory cytokines, leading to OA development. Thus, to determine the key genes in SFs involved in OA occurrence, it is important to identify subpopulations of cells and associated genes in OA development. RNA sequencing (RNA-seq) and single-cell RNA sequencing (scRNA-seq) could be very useful to fulfill this task. Through these approaches, we may be able to identify key genes associated with OA initiation and progression.

scRNA-seq data of SFs in OA in the GEO database were analyzed. In these studies, the genes and pathways associated with OA development were analyzed and identified by bioinformatics methods. In one of these studies, scRNA-seq data of SFs in OA patients were subjected to bioinformatics analyses, and OA-related genes were identified as those encoding ECM proteins and immune and cell adhesion molecules. Fibronectin 1 was identified as a key protein with functional changes during OA development.^177^

One comprehensive study was conducted using human OA cartilage at different stages to analyze the single-cell profiles of OA chondrocytes. This study shows a transition from proliferating chondrocytes to prehypertrophic and hypertrophic chondrocytes (HTCs) and identified a new subpopulation of cells among HTCs. The researchers also identified novel marker genes for cartilage progenitor cells and showed the association of these progenitor cells with fibrocartilage chondrocytes through bioinformatics analysis.^178^ A recent scRNA-seq study demonstrated that two marker genes (Col6a3 and ACTG1) were highly expressed in synovial tissues and were involved in focal adhesion. These two genes were upregulated during OA development, suggesting their roles in OA progression.^179^

## Lineage tracing

**Fig. 4:**
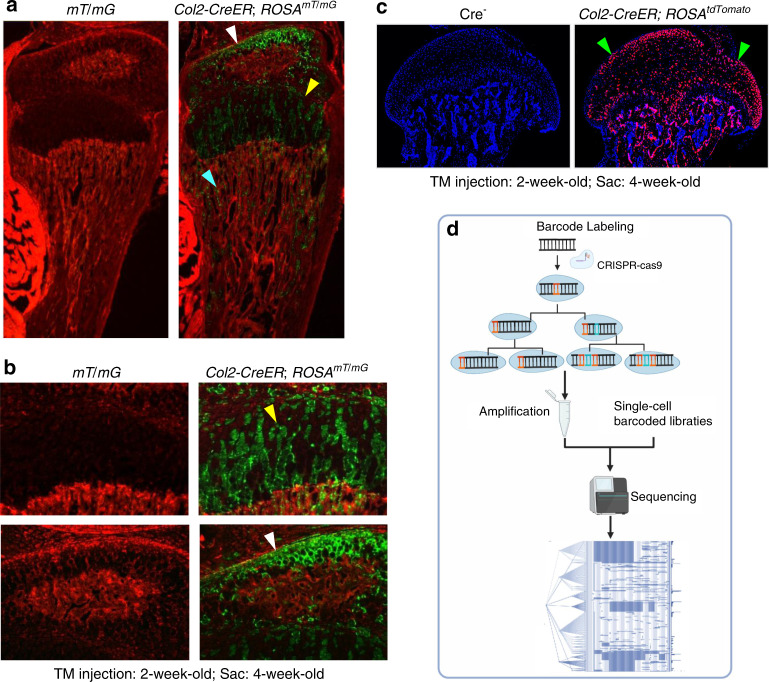
Lineage tracing technique. **a**–**c** Col2-expressing cells label a subpopulation of chondrocytes which could migrate to bone marrow underneath the growth plate and transdifferentiate into progenitor cells. **a** Col2-CreER mice were generated and bred with ROSA^mT/mG^ reporter mice or ROSA^tdTomato^ reporter mice. **b** Tamoxifen was intraperitoneally injected to the 2-week-old Col2-CreER; ROSA^mT/mG^ mice or Col2-CreER; ROSA^tdTomato^ mice for 5 consecutive days (1 mg per 10 g body weight) and mice were sacrificed at 4-week-old. **c** Col2-expressing cells were detected in articular chondrocytes, growth plate chondrocytes and cells located in bone marrow underneath the growth plate. **d** DNA barcoding technology was used for cell lineage tracing. Figure 4a, b^256^ and Fig. 4c^257^ were cited from our previous publications.

Lineage tracing has been successfully used in skeletal development studies. Several mouse models, such as *Osx-CreER* mice, *Col2-CreER* mice and *Nestin-CreER* mice, have been generated and used in lineage tracing studies. These mice were bred with ROSA26R-LacZ reporter, ROSA^tdTomato^ or ROSA^mT/mG^ reporter mice. The differentiation and migration of mesenchymal progenitor cells could be monitored by examining reporter activity and the fates of stage-selective subsets of osteoblast lineage cells. These studies revealed that perichondrial precursors, *Osx*-expressing cells or *Col2*-expressing cells (Fig. 4a–c) could migrate and differentiate into trabecular osteoblasts, osteocytes, and stromal cells inside the developing bone, and some of these cells were also associated with invading blood vessels and pericytes.^180^ These cells can maintain their progenitor cell phenotype into late postnatal life.^181^ Based on lineage tracing studies, we also learned that resting zone chondrocytes in the growth plate could migrate into the bone marrow underneath the growth plate and dedifferentiate into mesenchymal progenitor cells. These cells could further differentiate into osteoblasts, which are involved in bone formation.^182^ In addition, these dedifferentiated progenitor cells could also differentiate into adipocytes and regulate osteoclast formation.^183,184^ Although lineage tracing techniques have been successfully used in skeletal development studies, we know very little about how mesenchymal cells differentiate into synovial cells and articular chondrocytes under normal physiological or osteoarthritic conditions.

However, current lineage tracing approaches poorly reflect whole and complex organisms. In the past decade, the emergence and development of gene editing and DNA barcoding technologies have brought several advantages for cell lineage tracing^185,186^ (Fig. 4d). In 2016, McKenna and colleagues performed a combination of CRISPR/Cas9-based genome editing, DNA barcoding, and single-cell sequencing to read cell lineage information for the first time.^187^ This technique has been called ‘dynamic lineage tracing’.^188,189^ First, this approach marks individual cell lineages by inserting a compact DNA sequence into the genome. Then, the recombinase or CRISPR system is delivered or activated in cells to trigger barcode modification to increase the diversity of barcode sequences in cells during development. Finally, the barcode information is collected and analyzed to reconstruct a cell lineage tree by single-cell sequencing.^190^

## Cell death regulation

**Fig. 5:**
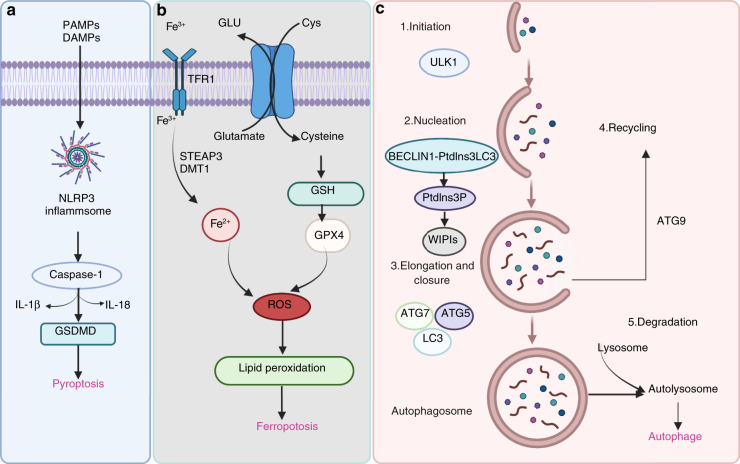
Pathways of pyroptosis, ferroptosis, and autophagy. **a** Many factors trigger assembly of the NLRP3 inflammasome, followed by activation of caspase-1 or caspase-11/4/5, which cleaves the gasdermin D (GSDMD) protein and pro-IL-1β and pro-IL-18, resulting in the release of IL-1β and IL-18. **b** During ferroptotic cell death, intracellular Glu is transported to the extracellular space, and extracellular Cys2 is transported into the cell, where it is then transformed into Cys for GSH synthesis. GPX4 reduces ROS accumulation. Excess iron is the basis for ferroptosis execution. Circulating iron binds with transferrin in the form of Fe^3+^ and then enters the cells by TFR1. Fe^3+^ is deoxidized to Fe^2+^ by the iron oxide reductase STEAP3. Ultimately, Fe^2+^ is released into a labile iron pool in the cytoplasm from the endosome via DMT1. **c** The canonical formation of autophagosomes involves the following steps: initiation, nucleation, elongation, closure, and recycling

Cell death is a fundamental physiological process in multicellular organisms that maintains homeostasis during embryonic development and responds to harmful environmental stimuli to eliminate superfluous, damaged, senescent, and potentially harmful cells.^191,192^ In contrast, programmed cell death is regulated by a complex and delicate genetic process and is beneficial for tissue homeostasis and immune responses.^193,194^ For many years, apoptosis has long been considered the only form of regulated cell death, and necrosis was considered an unregulated cell death process. In the past two decades, the existence of new forms of cell death has been established. Several new forms of regulated cell death, such as pyroptosis, necroptosis, ferroptosis, parthanatos, mitochondrial permeability transition (MPT)-dependent necrosis, autophagy, and pyronecrosis, have been identified and characterized.^195,196^ Recent findings have provided new insights into the mechanisms of regulated cell death and in vivo relevance to OA pathology and suggest that regulated cell death could have a significant impact on OA pathology (Fig. 5).

### Pyroptosis

Pyroptosis was identified by D’Souza et al. in 2001 and described as proinflammatory programmed cell death, which is different from apoptosis.^197^ This process induces cell lysis and causes the massive release of cellular contents and proinflammatory factors, contributing to the development of many autoimmune and inflammatory diseases. Pyroptosis is triggered by the inflammasome-driven activation of caspase-1 or caspase-11/4/5, which cleaves the gasdermin D (GSDMD) protein,^198–200^ separating its N-terminal pore-forming domain (GSDMD-NP) from the C-terminal repressor domain (GSDMD-CR).^201,202^ GSDMD-NP binds to the inner acidic phospholipids of the plasma membrane and forms pores in the plasma membrane, resulting in the release of IL-1β and IL-18. These proinflammatory cytokines are then processed by caspase-1 and induce pyroptotic cell death (Fig. 5a).

Excessive inflammation in chondrocytes plays a vital role in chondrocyte survival, leading to OA pathology. One study demonstrated that acid-sensitive ion channel 1a mediates chondrocyte pyroptosis in an adjuvant-induced arthritis model in rats.^203^ Another study revealed that NLRP3 was activated in an experimental OA model, and the NLRP3 inhibitor CY-09 protected against OA development.^204^ Our previous studies have demonstrated that loganin and morronside attenuate OA development by inhibiting chondrocyte pyroptosis.^26,205^ As the main effector cells of synovial fibrosis in knee OA, fibroblast-like synoviocyte pyroptosis was reported to be mediated by NLRP1 and NLRP3 inflammasomes in knee OA, and an increase in HIF-1α may exacerbate synovial fibrosis by promoting fibroblast-like synoviocyte pyroptosis.^206^ The molecular mechanisms underlying pyroptosis in OA need to be further investigated.

### Ferroptosis

Ferroptosis is a novel form of programmed cell death that is driven by iron-dependent lipid peroxidation but is morphologically and biochemically different from other types of regulated cell death.^207,208^ Dixon et al. first reported and named this nonapoptotic cell death, which is characterized by aberrant accumulation of lipid reactive oxygen species (ROS), in 2012.^209^ Ferroptosis can be regulated by different metabolic pathways, including redox and iron homeostasis, mitochondrial activity and the metabolism of amino acids, lipids, and sugars.^210,211^ GPX4 is the key regulator of ferroptosis. Ultimately, GPX4 inactivation results in iron-dependent intracellular accumulation of lethal levels of lipid hydroperoxides (Fig. 5b).

Many pathophysiological processes are involved in the induction of ferroptosis, such as neurodegeneration, ischemia/reperfusion injury, and stroke.^212,213^ In OA, inflammation and metabolic factors are two important contributors associated with OA cartilage loss and disease symptoms.^214,215^ A recent report demonstrated that chondrocyte ferroptosis was involved in OA progression.^216,217^ It has been demonstrated that the induction of chondrocyte ferroptosis increases MMP13 expression while decreasing collagen II expression. Intra-articular injection of ferrostatin-1, a ferroptosis inhibitor, prevented OA progression. Interestingly, several signaling pathways related to OA could regulate ferroptosis. Hippo-Yap signaling increases ferroptosis in cancer cells.^218–220^ Energy stress-mediated AMPK signaling inhibits fatty acid synthesis and negatively regulates ferroptosis.^221,222^ Hypoxia signaling promotes ferroptosis by activating hypoxia-inducible factors (HIFs) and increasing the production of ROS.^212^ This evidence suggests that pharmacological modulation of ferroptosis may hold great potential for the treatment of OA.

### Autophagy

Autophagy is a highly conserved process that results in lysosomal degradation of bulk cytoplasmic contents, abnormal macromolecule aggregates, and excess or damaged organelles, which is essential for cellular homeostasis.^223,224^ In eukaryotic cells, autophagy often refers to macroautophagy, which is characterized by the generation of double-membraned vacuoles called autophagosomes, which sequester cytoplasmic components before delivering them to the lysosome for degradation. The canonical formation of autophagosomes involves the following steps: initiation, nucleation, elongation and closure and recycling (Fig. 5c). Each step depends on a unique molecular mechanism. The important components of the autophagy signaling pathway include the ULK1 complex, the BECLIN1-PtdIns3KC3-ATG14L complex, WIPIs, ATG12-ATG5, LC3-PE conjugation systems, and ATG9.^223^ Activating mTOR suppresses autophagy and Akt and AMPK signaling, and inhibiting mTOR promotes AMPK and p53 signaling.^225–227^ In addition, hypoxia, reactive oxygen species and nutrient and energy deprivation can generally activate autophagy.

It is well known that OA is an aging-related disease associated with the accumulation of damaged macromolecules, which leads to chondrocyte dysfunction and death. Previous studies have reported that reduced expression of ULK1, Beclin1, and LC3 was observed in human OA and aging-related and surgically induced OA in mice, indicating a deficiency in autophagy regulation in OA chondrocytes.^228^ Recent studies have demonstrated that autophagy activation protects against mitochondrial dysfunction in human chondrocytes and that loss of function of mTOR in cartilage tissue protects mice from developing OA by upregulating autophagy.^229,230^ Thus, the use of therapeutics targeting the autophagy signaling pathway is a potential strategy for OA treatment. Certain autophagy-enhancing drugs have been shown to attenuate OA cartilage degeneration. Local intraarticular injection of rapamycin reduces mTOR expression and delays articular cartilage degeneration in a DMM-induced murine model of OA.^42,231^ Currently, a phase III clinical trial (NCT02905799) is being conducted to determine the efficacy of resveratrol for OA treatment.

## Synovial lymphatics in osteoarthritis

Currently, there is no effective therapy for OA. The identification of new pathways and mechanisms responsible for the initiation and progression of OA will advance the development of new therapies for OA. Cartilage-derived catabolic factors accumulate in the synovium of OA joints, but how these catabolic factors are cleared is currently unknown.

### The synovial lymphatic system (SLS)

Lymphatic vessels (LVs) are composed of capillary and collecting vessels.^232^ Capillary LVs have a thin layer of lymphatic endothelial cells (LECs) that express LV endothelial hyaluronan receptor 1 and podoplanin, a transmembrane glycoprotein.^233^ Collecting LVs are covered with lymphatic muscle cells that have phenotypic features of both striated and vascular smooth muscle cells.^234,235^ However, a recent lineage tracing study revealed that popliteal lymphatic muscle progenitor cells are distinct from skeletal (Pax7^+^ and MyoD^+^) and vascular muscle progenitors (Prrx1^+^ and NG2^+^) during development and after postnatal Day 10 and are derived from a previously unknown Pax7^-^/MyoD^-^/Prrx1^+^/NG2^−^ muscle progenitor.^236^ Lymph cells are propelled by alternating contraction and relaxation of lymphatic muscle cells to draining lymph nodes (DLNs) and eventually to the venous circulation.^237,238^

Using a combination of near-infrared and indocyanine green lymphatic imaging^239–244^ to monitor LV contraction and clearance from joints to DLNs and immunofluorescence staining/whole slide imaging to identify and qualify LVs in mouse joints,^245,246^ we reported that LVs are present in the synovium and surrounding soft tissues in the knee joint, including the joint capsule, fat pads, ligaments, and muscles. Knees drain to iliac LNs (ILNs), while ankles (footpad) drain to popliteal LNs (PLNs) via collecting LVs.^241,245,247^ These LVs are therefore named the synovial lymphatic system (SLS), which consists of initial LVs in the synovium and the surrounding soft tissues, DLNs, and collecting LVs (large vessels whose contractions move lymph from initial LVs to DLNs).^248^

### The SLS in arthritis

The role of the SLS in murine and human rheumatoid arthritis (RA), which is an autoimmune disease, has been actively studied in the past 15 years, and the results reveal its importance in RA progression and treatment (for details please see the review).^248^ This conclusion is based on several reports. (a) In RA mouse models, including collagen-induced arthritis mice, KRN transgenic mice and TNF transgenic mice, SLS draining correlates with the progression of joint damage and is improved by drugs that reduce RA pathology. (b) Stimulation of lymphangiogenesis by vascular endothelial growth factor C (VEGF-C) and its receptor (VEGFR3) attenuates RA joint damage, and inhibiting lymphangiogenesis with a VEGF inhibitor accelerates RA joint damage. VEGF-C is considered a lymphatic-specific growth factor because VEGFR3 is mainly expressed by LECs. (c) More recently, altered LV anatomy and markedly decreased lymph clearance were observed in the affected hands of individuals with active RA,^249^ providing strong evidence for the involvement of the SLS in human RA. OA patients suffer from similar joint stiffness and pain as RA individuals, but these types of arthritis are very different in origin. OA is associated with long-term mechanical wear and tear on the cartilage during natural aging, while RA is an autoimmune disease. The involvement of the SLS in human and murine RA has been well studied; however, only a few reports have focused on the SLS in OA.

#### SLS studies in OA

Early case studies reported that a radiolabeled tracer was cleared to DLNs after being injected into a normal joint, and it accumulated in the synovial space following injection into an OA joint of the same individual.^250^ These findings suggested that the injected tracer was removed from the synovial space via LVs in normal joints and that this process was impaired in OA joints.

A more systemic clinical study was performed by immunostaining 60 knee samples taken from severe knee OA patients during total knee replacement surgery. Sixty postmortem control knees were stained with podoplanin, a commonly used marker for LECs, and LV density and LEC fractional area were quantified. Similar results were obtained as those found in a mouse study.^245^ LVs were detected in synovial tissue but not in subchondral bone. OA samples exhibited lower LV density and fewer LEC fractional areas than nonarthritic controls. In individuals with OA, low LV density and LEC fractional areas were associated with clinically detectable effusion but not with radiological or histological severity of OA tissue damage. These findings support a pathogenic role of synovial drainage via LVs in effusion as a clinical sign of synovitis in OA.^251^

#### SLS studies in OA mouse models

To investigate whether the SLS contributes to OA pathology and identify the potential mechanisms involved, we examined lymphatic drainage in WT mice with posttraumatic OA (PTOA) and found that lymphatic drainage was decreased in PTOA joints.^245^ VEGFR3-neutralizing antibody administration further reduced synovial lymphatic drainage and accelerated joint tissue damage. Synovial LECs that were isolated from PTOA joints based on podoplanin expression had increased expression of inflammatory factors. Interestingly, more macrophages, mainly M1 macrophages, were located adjacent to podoplanin^+^ LVs, and these M1 macrophages stimulated inflammatory gene expression in LECs in cocultures. Intra-articular injection of bortezomib increased lymphatic drainage and decreased the number of M1 macrophages in synovial tissue, resulting in decreased cartilage loss. Thus, local joint delivery of bortezomib or other anti-inflammatory drugs may restore synovial lymphatic function in individuals with posttraumatic knee OA.^25^

OA develops primarily as a result of aging. Interestingly, lymphatic dysfunction in aging has been recognized recently. In 2018, Mesquita et al. reported that in the brain, meningeal LVs remove cerebrospinal fluid macromolecules. Disruption of meningeal LVs in mouse models of Alzheimer’s disease promoted amyloid deposition and exacerbated parenchymal amyloid accumulation.^252^ Treatment of aged mice or mice with Alzheimer’s disease with VEGF-C enhanced meningeal lymphatic drainage. This study also showed decreased VEGFR3 signaling in meningeal LECs isolated from aged mice. Thus, it is likely that VEGF-C/VEGFR3 downregulation represents a new underlying mechanism for age-related lymphatic dysfunction.

### Arthritogenic matrix metalloproteinase (MMP13) is cleared from arthritic joints via the SLS

**Fig. 6:**
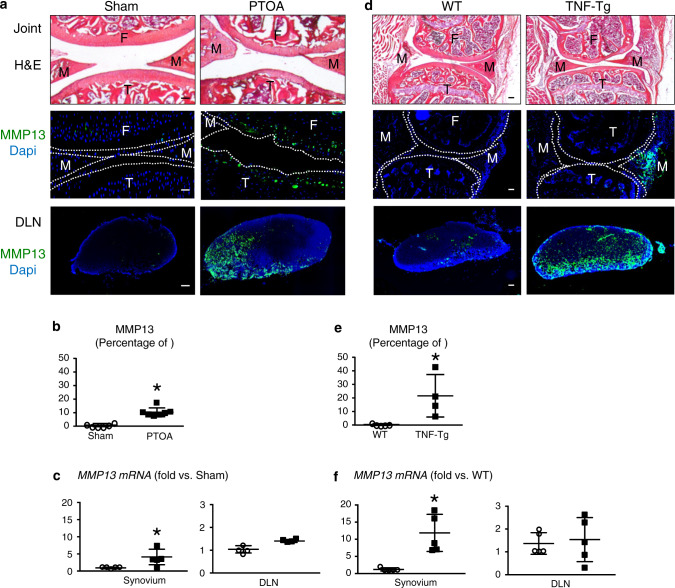
Arthritogenic MMP13 is removed from osteoarthritic and inflamed joints via the synovial lymphatic system. C57BL/6J mice received sham or DMM surgery (**a**–**c**), and TNF-Tg and WT control mice (**d**–**f**) were used. Knees and DLNs were harvested from operated mice 6 weeks post-surgery (**a**–**c**) and from 6-month-old WT and TNF-Tg mice (**d**–**f**). **a**, **d** Frozen sections from the knees were H&E stained for light microscopy, and representative 10x images are shown (scale bar = 100 µm). F: femur, T: tibia, M: meniscus. Parallel knee sections (dotted lines indicate the joint space) and DLN sections were immunostained with green fluorescent labeled antibodies against MMP13, counterstained with DAPI, and representative dark field images obtained at 10x are shown. Note that only articular chondrocytes in PTOA knees, synovium in TNF-Tg knees, and their DLNs, have robust MMP13 immuno-reactivity. **b**, **e** VisioPharm histomorphometry was performed to quantify the percentage of MMP13^+^ area of the DLN immunostained sections, and data are presented for each DLN +/− SD (*P < 0.05 vs. non-arthritic control with paired *t*-test). **c**, **f** *Mmp13* mRNA levels in knees were assessed via qPCR. The data are presented for each tissue ± SD (*P < 0.05 vs. non-arthritic control with paired *t*-test). The content presented in Fig. 6 was derived from a PhD dissertation and was provided by Dr. Xi Lin, with her permission, a postdoctoral fellow in University of Rochester Medical Center^258^

The SLS drains large molecules to DLNs. MMP13 is a critical catabolic enzyme in both RA and OA arthritic joints that destroys cartilage. Despite some beneficial effects on arthritic models,^253^ MMP13 inhibitors have shown no greater advantages than placebo in OA clinical trials, and notable musculoskeletal toxicity, such as arthralgia and edema, was observed.^254^ To examine whether the MMP13 protein can be cleared from arthritic joints to DLNs via the SLS, we examined MMP13 protein and gene expression in the synovium and corresponding DLNs by immunohistochemistry (IHC) and RT–qPCR in PTOA or TNF transgenic (TNF-Tg) mice. MMP13 protein and mRNA expression was significantly higher in the synovium of PTOA and RA mice than in controls. MMP13 protein levels in the DLNs of arthritic mice were also markedly upregulated compared to those of control mice, but no differences in mRNA levels were detected (Fig. 6). The IHC results showed that MMP13 was expressed on only articular chondrocytes in mice with PTOA and the synovium in TNF-Tg mice (Fig. 6a, d). Consistently, the DLNs in these two established mouse models exhibited robust immunoreactivity, while no MMP13 expression was detected in the DLNs of control mice without arthritis (Fig. 6a–e). Interestingly, we only detected the expression of *Mmp13* mRNA in the knee synovium but not in DLNs (Fig. 6c, f). Given the high MMP13 protein expression in DLNs without *de novo* synthesis, these data demonstrate that matrix metalloproteinases expressed in the DLNs came from the afferent arthritic knee and was translocated via SLS clearance. Furthermore, the SLS plays an important role in the efficient clearance of catabolic large molecules that lead to cartilage breakup, which is critical for protecting against arthritic damage. Thus, improving lymphatic clearance is a viable strategy for arthritis treatment.

Most SLS studies are focused on the association between the SLS and arthritis during disease progression and the response to treatment. For example, SLS dysfunction becomes more severe with arthritis progression, and drugs that attenuate joint damage are often associated with the restoration of SLS function. In these studies, the specific involvement of LVs in arthritis is evidenced by LEC-specific manipulation, including the use of VEGF-C- and VEGFR3-neutralizing antibodies or signaling inhibitors. Since the VEGF-C/VEGFR3 signaling pathway affects processes than those of LECs, such as macrophage activation,^255^ using a genetically modified mouse model with LEC-specific manipulation is needed to provide more direct evidence of the causal role of the SLS in arthritis pathogenesis. Currently, a major mechanism for SLS dysfunction in RA and OA is associated with inflammation. In synovial tissue, inflammatory factors and cells such as macrophages can affect LV cell function. Furthermore, LV cells, including LECs and lymphatic muscle cells, can be inflamed, resulting in functional changes. It will be very interesting to identify molecular signatures of LECs and lymphatic muscle cells in an arthritic microenvironment and discover new pathological genes/pathways that are intrinsically expressed by LV cells.

## Conclusions

Over the past decades, our understanding of OA pathogenesis has expanded from OA being a ‘wear and tear’ disease to whole joint pathology featuring synovitis, cartilage damage, subchondral bone remodeling, and osteophyte formation. OA pathology involves a variety of factors, such as mechanical loading, aging, inflammation and metabolic changes, and the activation of different signaling pathways, such as Wnt/β-catenin, Ihh, TGF-β, EGFR, HIF, NF-κB, and Notch. New approaches and novel techniques have been explored and developed in OA research from different aspects. Epigenetic regulation, such as DNA methylation, histone modification, and miRNA regulation, provides new insights into the pathogenesis of OA at the transcriptional and/or posttranscriptional level. A variety of cell death types have been observed in OA development and have significant impacts on OA pathology. The synovial lymphatic system plays an important role in the clearance of cartilage-derived catabolic factors in the synovium of OA joints, which opens up novel research directions. Novel techniques, such as CRISPR/Cas9 genome editing, single-cell RNA sequencing, and lineage tracing, have been developed and used in OA studies, and these techniques greatly facilitates the discovery of new methods and drug candidates for OA treatment. Despite the significant progress in our understanding of OA pathogenesis, the etiology and pathological mechanisms of OA are not yet fully understood. It is conceivable that the application of novel techniques could accelerate our understanding of OA pathogenesis. In the future, it will be crucial to explore the molecular mechanisms underlying OA-associated pain and articular pathology as well as the relationships between them, which would be helpful in developing more specific and effective therapeutic interventions for OA.
